# Microbial communities associated with two populations of the sponge *Chondrilla nucula* under present and projected climate conditions in the Aegean Sea

**DOI:** 10.3897/BDJ.14.e187301

**Published:** 2026-04-29

**Authors:** Anastasia Gioti, Jon Bent Kristoffersen, Bekir Kaşlı, Georgia Tarifa, Carmen Rizzo, Thanos Dailianis

**Affiliations:** 1 Hellenic Centre for Marine Research, Institute of Marine Biology, Biotechnology and Aquaculture, Heraklion, Greece Hellenic Centre for Marine Research, Institute of Marine Biology, Biotechnology and Aquaculture Heraklion Greece; 2 Ludwig Maximilian University Munich, Munich, Germany Ludwig Maximilian University Munich Munich Germany; 3 Stazione Zoologica Anton Dohrn, Sicily Marine Centre, Messina, Italy Stazione Zoologica Anton Dohrn, Sicily Marine Centre Messina Italy

**Keywords:** Porifera, 16S, climate change, metabarcoding, Mediterranean, ocean warming, ocean acidification, common-garden experiment, Fungi, Oxford Nanopore Technology, Illumina, 18S, ITS, 28S, *Chondrilla nucula*

## Abstract

This data paper describes bacterial and fungal communities associated with the sponge *Chondrilla nucula* collected from two Eastern Mediterranean populations (North and South Aegean Sea) and maintained under controlled common-garden conditions simulating present and projected climate scenarios over a period of 3 months. Microbial composition was characterised using two complementary ribosomal marker approaches: Illumina (MiSeq) sequencing of the 16S rRNA gene for Bacteria and Oxford Nanopore (MinION) sequencing of a long 18S-ITS-28S rRNA fragment for Fungi. A total of 24 sponge libraries (3 climate conditions x 2 populations x 4 biological replicates) along with six control libraries (water from three experimental tanks, extraction and PCR blanks) were constructed for each group of microsymbionts. The resulting reads were processed using custom and publicly available bioinformatic pipelines and databases, followed by initial taxonomic assignment. This dataset represents the first fungal community associated with *C. nucula* and the first bacterial community for this species from the Aegean Sea.

## Introduction

Microbes living in association with marine animals or plants often exhibit greater genomic plasticity and faster adaptive responses than their hosts, suggesting that the symbiotic microbiome may play critical roles in host survival under future climate conditions (Bang et al. 2018). These roles are especially relevant for sessile organisms such as sponges, which have limited capacity to escape environmental stress. Sponges host diverse and structured microbial communities that are species-specific (Dittami et al. 2019) and contribute to host physiology through nutrient cycling, vitamin biosynthesis and production of defensive metabolites (Webster and Thomas 2016). Microbial contributions to host acclimatisation and resilience under stress are less understood; shifts in microbial communities have been reported under marine heatwaves and ocean acidification (e.g., Efremova et al. (2024), Bell et al. (2024)), but it remains difficult to disentangle natural environmental variability from responses to specific stressors. Controlled experimental approaches on the topic remain relatively scarce (Fan et al. 2013, Ramsby et al. 2018).

The Mediterranean Sea is a hotspot of marine heatwaves (Frölicher et al. 2018), driving mass mortality events that strongly affect sponges (Garrabou et al. 2019). Characterising the microbiomes of sponge populations across natural thermal gradients may help identify potential source populations and microbial reservoirs associated with resilience (Núñez-Pons et al. 2025). *Chondrilla nucula* (Porifera, Demospongiae) is a common resident of the Aegean Sea (Voultsiadou 2005) and an excellent model for studying responses to climate-related stressors due to its innate preference for shallow habitats that are particularly exposed to environmental fluctuations (Usher et al. 2004). Knowledge of its microbiome remains limited to two studies conducted before the advent of next-generation sequencing (Thiel et al. 2007, Chelossi et al. 2007) and a recent one focused on its functional association with *Posidonia oceanica* (Berlinghof et al. 2025). No study to date has characterised the eukaryotic microbial community of *C. nucula*, although vertically-transmitted yeasts were microscopically detected in Caribbean populations (Maldonado et al. 2005).

Fungi represent a largely overlooked component of biodiversity (Phukhamsakda et al. 2022). Little is known on their interactions with the marine biosphere (Amend et al. 2019) and their responses to climate change (Bahram and Netherway 2021), despite evidence that ocean acidification may favour their dispersal (Yarden 2014). The study of fungal diversity is further complicated by limitations of the universal fungal ITS barcode (Schoch et al. 2012) in capturing divergent and novel lineages (Lindahl et al. 2013). Long-read sequencing appears promising for a more accurate taxonomic placement of Fungi, since it allows increasing the number of genes (Tedersoo et al. 2022). The approach is currently limited by the reduced representation of Fungi in public databases.

In order to assess the potential responses of the *C. nucula* microbiome to climate change, we studied the prokaryotic and fungal communities associated with Aegean Sea sponges that were subjected to a controlled common-garden experiment simulating future temperature and pH conditions.

### Value of the dataset

This dataset enables: (i) Analysis of microbial community dynamics of sponges under controlled climate-change stressors (warming and acidification); (ii) Comparison of microbiomes from populations adapted to different natural thermal regimes within the same species. Furthermore the study represents: (iii) The first comprehensive reference of the prokaryotic and fungal communities of *Chondrilla nucula* in the Aegean Sea; (iv) A baseline for future monitoring of sponge-associated microbial communities in the region; v) A methodological baseline for identifying fungal taxa from marine organisms using long-read sequencing.

## Methods

Here, we detail sampling design, laboratory procedures and bioinformatic processing of the sequencing data, along with the taxonomic structure of microsymbiont communities as derived from initial, unfiltered abundance tables for the identified amplicon sequence variants (ASVs) / consensus sequences.

### Acquisition of samples and experimental treatment

Specimens of *C. nucula* were collected at ≤5 m depth from wild populations in the South and North Aegean Sea. They were transferred to experimental aquaria, acclimatised for 40 days and maintained in a controlled setup simulating present-day conditions and the projected high greenhouse gas emissions scenario (RCP 8.5). Specifically, three climate change scenarios were simulated: 1) the Control, in which the ambient temperature was estimated as the average of the maximum summer temperatures (27°C) between the two populations (north and south Aegean). The pH was ambient (~ 8.1); 2) the South Aegean Climate Change (SACC), in which temperature was estimated as the maximum recorded in South Aegean during summer (27°C) increased by 4°C. The pH was decreased by 0.3 units (~ 7.8); and 3) the North Aegean Climate Change (NACC), in which temperature was estimated as the maximum recorded in North Aegean during summer (26°C) increased by 4°C. The pH was decreased by 0.3 units (~ 7.8). The experiment is detailed in Chatzinikolaou et al. (2026). At the end of the three-month period, a group of four individuals from each population per treatment were randomly sampled, gently cleaned of debris and frozen at -80°C prior to DNA extraction. Water samples (10 lt) from each of the three experimental tanks were collected in sterile plastic bottles and stored at -20°C due to time limitations to directly process them.

#### Geographic range

Two locations in Greece were used for sponge sampling: 1) North Aegean Sea, Chalkidiki Peninsula (latitude: 39.9315, longitude: 23.7348); 2) South Aegean Sea, Hersonnissos Village (latitude: 35.3301, longitude: 25.3872).

### Sample processing

**DNA extractions**

High-molecular weight DNA (n = 24) was extracted from tissue fragments ground with liquid nitrogen in sterile mortars and pestles. The powder served as input for the DNA extraction, using the Quick-DNA™ Fungal/Bacteria kit (ZymoResearch, USA) according to the manufacturer’s instructions with the following modifications: 1) added b-mercaptoethanol in genomic lysis buffer; 2) cells were lyzed for 5 min at 30 Hz in the Tissuelyser apparatus; 3) performed an additional centrifugation before elution; 4) eluted two times in 20 ul elution buffer (10 mM Tris, pH 8.5, 0.1 mM EDTA) with 3 minutes waiting in between elutions.

The same procedure was used for the negative control of the ZymoResearch extraction / extraction blank (sample: "EB"), where sterile water was used as input material. DNA for aquarium water metagenomes (n = 3) was extracted as follows: the plastic bottles containing water were put at room temperature to de-freeze and their content was filtered with 0.22 μm Sterivex filters using a peristaltic pump. DNA was then extracted with the PowerWater Dneasy Kit following the manufacturer's protocol, except for the addition of a 10-minute incubation step at 65° and two additional washes; the DNA was eluted in 100 ul of a 10 mM Tris solution. The same procedure was followed for the negative control of the filtering procedure (sample: "Negative_new"), where sterile water was filtered through the peristaltic pump and for the 2^nd^-step PCR control (sample: "PCRblank"), where sterile water was used as input for the amplification step.

**Prokaryotic ribosomal gene amplification and Illumina MiSeq sequencing**

**Figure 1.**
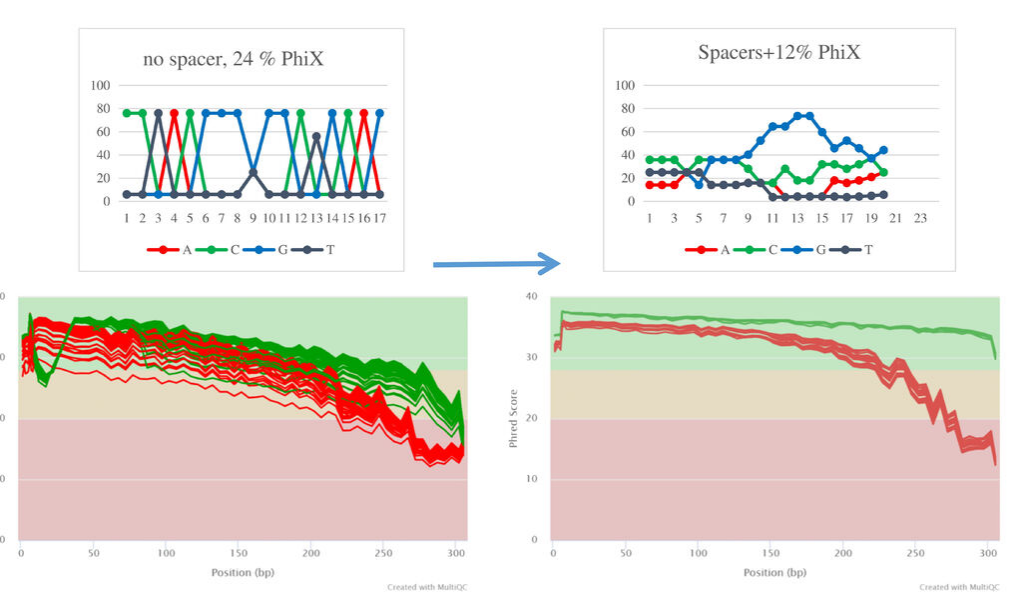
Improvement of base diversity (top panel) and quality (bottom panel) in Illumina MiSeq sequencing with the introduction of spacers. Read 1 data from two runs of 16S rRNA libraries were analysed: one constructed without spacers (left panel, unpublished data) and the second with spacers (right panel, current experiment). Protocols, reagents and instruments were identical and were carried out at the same sequencing unit by the same technician. Base diversity plots in ideal conditions would show all bases close to 25% in each cycle, while base quality plots would show Phred scores exceeding 20 in all positions.

A two-step, combinatorial dual index PCR protocol was used to amplify the hypervariable V3-V4 region of the 16S rRNA gene. The primers 341FB (Klindworth et al. 2013) and 805RB1, based on Apprill et al. (2015) and further modified by Pavloudi et al. (2017) were modified with diversity spacers (Suppl. material 1) as in Holm et al. (2019) to improve the library diversity and the resulting sequence quality (Fig. 1). The 1^st^-step PCR used primers with a TruSeq-compatible tail. The reaction contained 120 ng of template DNA, 5x KAPA Fidelity buffer at a final concentration of 1x; dNTPs at 0.3 mM; forward primer mix at 0.3 uM, reverse primer mix at 0.3 uM, KAPA HiFi Hotstart with dNTPs polymerase (Roche, Switzerland) at 0.02 Units/ul and ddH_2_O up to a total volume of 24 ul. The cycling conditions were: Initial denaturation at 95°C for 5 min, followed by 25 cycles of 98°C for 20 s, 58°C for 45 s, 72°C for 45 s and a final extension at 72°C for 7 min. The PCR products were purified with Nucleomag NGS Cleanup and Size-Select magnetic beads (Macherey-Nagel, Germany) with a bead: DNA ratio of 0.91:1.

The second-step PCR primers, based on Glenn et al. (2019), were modified to include one phosphorothioate bond at the 3’ end, reported to protect the primers from 3' exonuclease activity of proof-reading DNA polymerases (Gohl et al. 2021). The PCR reaction contained 2 ul of 1^st^-step PCR product, 5x KAPA Fidelity buffer at a final concentration of 1x; dNTPs at 0.3 mM; forward and primer at 1 uM, KAPA HiFi Hotstart polymerase at 0.025 Units/ul and ddH_2_O up to a total volume of 24 ul. The cycling conditions were: Initial denaturation at 95°C for 3 min, followed by 8 cycles of 98°C for 30 s, 55°C for 30 s, 72°C for 30 s and a final extension at 72°C for 5 min. The PCR products were purified with Nucleomag NGS Cleanup and Size-Select magnetic beads, with a bead: DNA ratio of 0.8:1. The purified products were gel size-selected (main band: ~ 600 bp) with Macherey-Nagel NucleoSpin Gel and PCR Clean‑up and purified with a magnetic bead cleanup with a bead: DNA ratio of 0.9:1. They were quantified with a Qubit fluorometer (Thermo Fisher Scientific, USA) and pooled in equimolar amounts. The library pool was quantified by qPCR with a NEBNext Library Quant Kit for Illumina (New England Biolabs, USA) and sequenced on an Illumina MiSeq sequencer in paired-end mode (2*306 cycles). Assessment of read quality, length and relevant quality metrics was performed with FastQC. One sample (NACC scenario, North population, bioreplicate 3) was excluded from final analysis at this stage.

**Εukaryotic ribosomal gene amplification and Oxford Nanopore sequencing**

Amplification of the eukaryotic ribosomal gene was performed using the primer pair SR1R:5’-TACCTGGTTGATYCTGCCAGT-3’ and LR11: 5’-GCCAGTTATCCCTGTGGTAA-3’ (Vilgalys and Hester (1990), Vilgalys laboratory primers) which spans almost the entire 18S-ITS-28S region (approximately 5.5 Kb). The PCR reaction contained 100 ng of template DNA, 5x Phusion HF buffer at a final concentration of 1x; dNTPs at 0.2 mM; forward and primer at 0.5 uM, Phusion Taq polymerase (ThermoFisher Scientific, USA) at 0.02 Units/ul and ddH_2_O up to a total volume of 50 ul. The cycling conditions were: initial denaturation at 98°C for 30 sec, followed by 25 cycles of 98°C for 10 s, 62.4°C for 30 sec, 72°C for 2.75 min and a final extension at 72°C for 10 min. Nanopore libraries were constructed following the Oxford Nanopore protocol for Native Barcoding kit V14, SQK-NBD114.96. To obtain the required 200 fmol (675 ng for a 5.5 Kb amplicon) input in a volume of 12 ul, the products of three PCR reactions, each 50 ul, were merged for each sample and concentrated by magnetic bead cleanup using NGS Clean-up and Size Select (Macherey-Nagel, Germany), with elution in 17 ul water. One sample ('t1p2r3_1st_2nd_ex') was sequenced with three different barcodes as technical replicates to investigate possible biases from using different library barcodes. To enable library quality assessment, 0.5 ul of CS DNA (a 3.6 Kb amplicon from the Lambda genome) was added to each sample as a positive control at the ligation step. Sequencing was performed using 140 ng of library input and the MinKNOW software v.23.07.12 (Oxford Nanopore Technologies, United Kingdom) on a R10.4.1 flow cell of the MinION device at the sequencing platform of the Institute of Marine Biology, Biotechnology & Aquaculture (IMBBC). Reads were basecalled using Dorado v.070 (Oxford Nanopore Technologies) with the 'sup' ('super accurate') model, with mandatory same barcode at both ends of the read and minimum quality score of 10. Demultiplexing, adapter and barcode trimming were performed with Dorado v.070 demux with default settings.

### Data processing

**Bioinformatic analysis of prokaryotic ribosome Illumina data**

**Figure 2.**
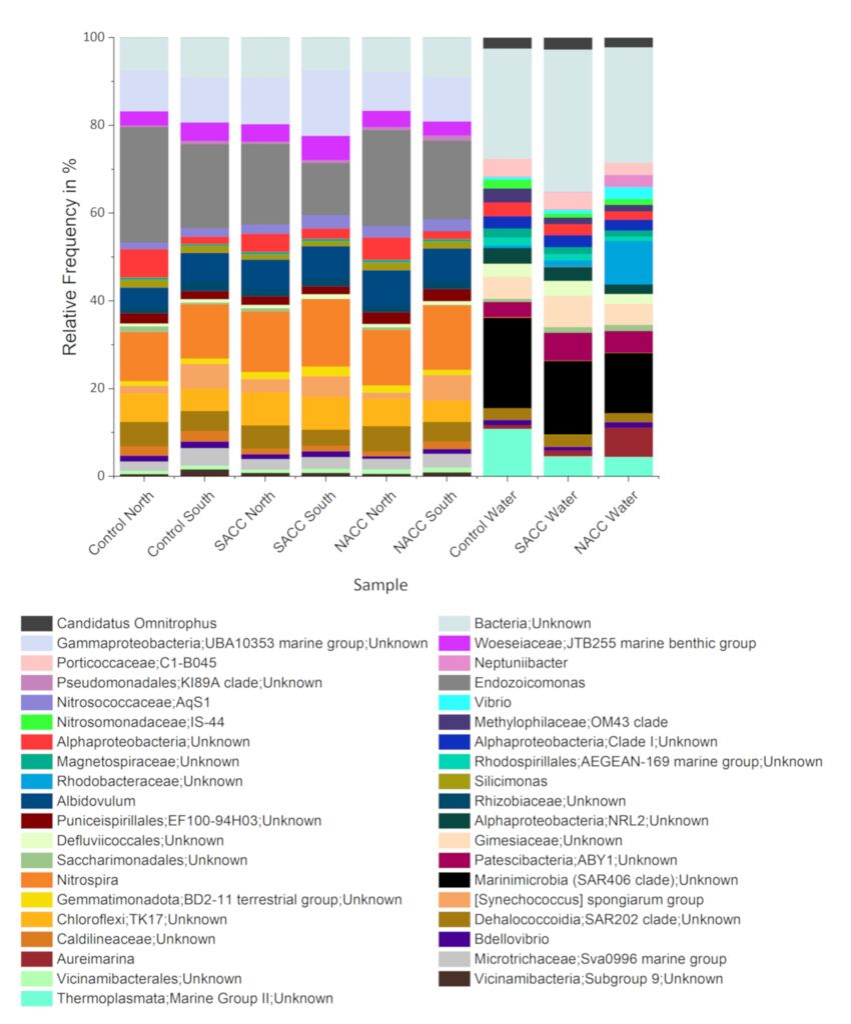
Genera of prokaryotes (Bacteria and Archaea) associated with *Chondrilla nucula* from two different geographic locations in the Aegean (North/South) under South Aegean Climate Change (SACC: 31°C, pH=7.8), North Aegean Climate Change (NACC: 30°C, pH = 7.8) and Control (27°C, pH = 8.1) treatments, as well as with water from the aquaria. The barplots present relative frequencies (%) per sample (calculated from abundance values averaged over replicates) for genera with relative abundance of over 1%. Plots were generated using OriginPro2028 software.

A total of 1.46 Million reads were obtained from all samples, with an average 540,036 reads for each read pair, excluding negative controls; these contained between 132 and 1,266 reads (Suppl. material 2). Primers and "read-through" (reads running into the reverse complement of the other primer) were searched and removed in both reads of each pair with cutadapt v.4.1 (Martin 2011). Next, we used the singularity version v.2.1.5 of the PEMA pipeline (Zafeiropoulos et al. 2020) at the high-performance computing bioinformatics platform at IMBBC (Zafeiropoulos et al. 2021) to further process reads and analyse their taxonomic content. Briefly, TruSeq3 adapters and low-quality bases were searched and removed using Trimmomatic (Bolger et al. 2014) by allowing three mismatches, setting to 30 the threshold for matching between the two 'adapter ligated' reads for PE palindrome read alignment, and to 5 the threshold for matching between any adapter sequence against a read. Trimming for low-quality bases involved the adaptive quality algorithm with parameters: targetLength = 305, strictness = 0.7 and removal of bases with quality below 10 and 20 from the beginning and the end of the read, respectively, as well as removal of reads below 70 bp. Resulting reads were then merged with PEAR (Zhang et al. 2014) with the minimum overlap between forward and reverse reads set to 5 bp, alignment quality threshold at 0.7 and elimination of all sequences with uncalled nucleotides in the output. ASVs were inferred through clustering with swarm v.2 (Mahé et al. 2015) and the maximum number of differences to group together two amplicons (d) set at 2. Alignment-based taxonomy assignment of ASVs was performed using the lowest common ancestor (LCA) Classifier algorithm within CREST (Lanzén et al. 2012) and the SILVA database v.1.3.8 (Chuvochina et al. 2025), using the default confidence value LCA range (x) of 2%. A total of 2,541 unique ASVs were classified at the genus level from the above analysis (Fig. 2).

**Bioinformatic analysis of eukaryotic ribosome Nanopore data**

Following dual-end demultiplexing, 3.15 million reads were assigned to the used barcodes; reads with quality score below 10 or not assigned to any barcode were removed. Sequencing error rates were estimated at 1% by mapping the reads to the known reference of CS DNA using minimap2 (Li 2018) following the author’s recommendations. The reads were processed with the PRONAME pipeline (Dubois et al. 2024). Briefly, at the proname_filter step, the primer set was removed with cutadapt (Martin 2011) and reads with length less than 1500 bp and quality score less than 15 were excluded (Suppl. material 3). Next, clustering was performed at a 0.99 id threshold with vsearch (Rognes et al. 2016) using the proname_refine script. Following polishing of consensus sequences (i.e. each cluster's centroid sequence) with medaka, we detected chimeras (0.15% of total reads) using a reference dataset (--chimeradb option) of 173,112 unique sequences, built by merging sequences from the following sources: i) the General Eukaryome Long database v.1.9.4, which contains long-read community-curated rRNA sequences (Tedersoo et al. 2024); ii) the rEGEN-B Bacteria database (15/01/2025 update); ii) the Fungal rRNA Operon Database for ONT-sequences (FRODO) (Lu et al. 2022); iii) all Porifera rRNA sequences, retrieved from the SILVA v.1.3.8 and the NCBI nucleotide database browsers and clustered with vsearch at a 99% sequence identity threshold. The high-quality consensus sequences generated by the proname_refine script were then processed with ITSx v.1.1 (Bengtsson‐Palme et al. 2013) using the -F parameter to extract the fungal SSU (18S), ITS (ITS1, 5.8S and ITS2 combined) and LSU (28S) regions as separate gene fragments.

**Figure 3.**
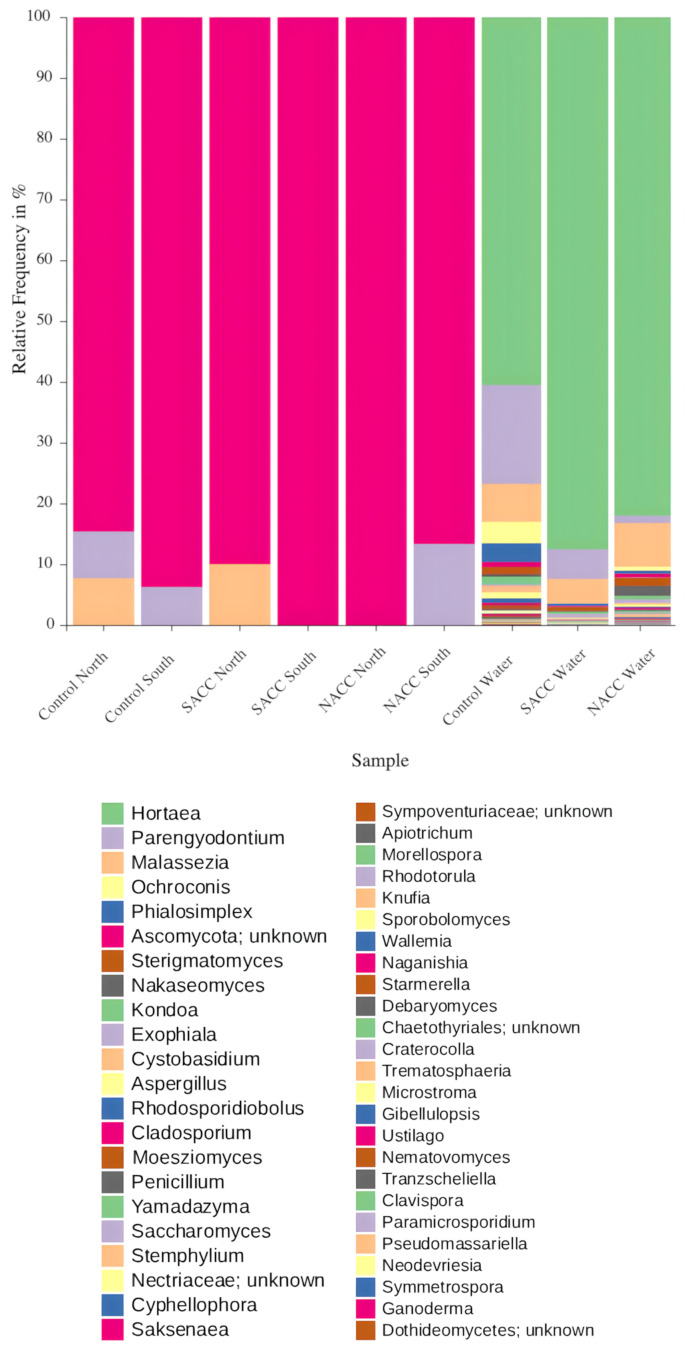
Genera of fungi associated with *Chondrilla nucula* from two different geographic locations in the Aegean (North/South) under South Aegean Climate Change (SACC: 31°C, pH = 7.8), North Aegean Climate Change (NACC: 30°C, pH = 7.8) and Control (27°C, pH = 8.1) treatments, as well as with water from the aquaria. The barplots present relative frequencies (%) per sample for genera classified with 0.8 or higher confidence. Plots were generated using the QIIME2view interface.

Classification was performed with the RDP Classifier release 2.14 (Wang et al. 2007), a k-mer–based probabilistic approach well-suited for rapid analysis of long-read data when combined with broad databases (Marić et al. 2024). Since the native training dataset shows low representation in fungi, we constructed eight custom training datasets and subjected these to different filtering steps to choose the most suitable for reliable classification of fungal ONT reads (Suppl. material 4). The datasets contained sequences spanning the 18S–ITS–28S ribosomal gene fragment (“LONG”), the 18S gene (“SSU”), the full ITS1-5.8S-ITS2 fragment (“ITS”) and the 28S gene (“LSU”). These sequences were derived from the Eukaryome database latest version (v.2.0), by applying an awk command on the fasta headers to retain those including the term “Fungi”; the LONG dataset further contained fungal sequences from FRODO. The datasets were curated to include only sequences that are fully classified until the genus level, for which the RDP classifier has been designed to provide taxonomies. To extend the classifier's taxonomic resolution, we compiled, for each of the four datasets, an additional species-level version (containing only sequences fully classified down to the species level). Intraspecific sequence variability, which affects the False Positive Rate (FPR) in k-mer based approaches (Graetz et al. 2025), was estimated by calculating unique taxa counts from the fasta header information using Biopython libraries (Cock et al. 2009). Based on this criterion, the species-level datasets were excluded due to comparatively low variation (Suppl. material 5A). Model performance was next evaluated (Suppl. material 5B), through Area Under the Curve (AUC) values, computed from Specificity and Sensitivity estimates, provided by the RDP cross-validation function. We employed the trapezoidal rule and the following formulae: Sensitivity = TPR = TP / (TP + FN) , 1- Specificity = FPR = FP / (FP + TN) , AUC = sum (i = 1 to n−1) (FPR (i+1) − FPR(i)) * (TPR(i+1) + TPR(i)) / 2, where TPR = true positive rate; FN = false negatives; TN = true negatives. From the above analyses, we selected ITS as the training reference set for RDP classification, since it achieved a FPR of 0.00 while maintaining a sensitivity of 0.93 at the 80% bootstrap threshold for genus assignments (Suppl. material 4). Using this cutoff, a total of 1,496 consensus sequences were classified with 0.8 or higher confidence value at the genus level (Fig. 3).

#### Technologies used

Illumina MiSeq sequencing, Οxford Nanopore MinIon sequencing, High-performance Computing

## Biodiversity target

Microbial prokaryotic and eukaryotic biodiversity in the marine environment.

### Taxonomic range

Bacteria, Fungi, Archaea

## Data Resources

Raw sequence data associated with the study are publicly accessible under the "umbrella" project PRJNA1422169. Eukaryotic ribosome ONT and prokaryotic ribosome Illumina data were deposited at the Sequence Read Archive and the European Nucleotide Archive (David et al. 2025) under Bioproject numbers PRJNA1415251 and PRJEB85273, respectively. Pending resolution of technical issues concerning accession runs ERR14231480, ERR14231482, ERR14231490 and ERR14231499, download links and metadata for all sequences of the present study are provided in Suppl. material 6.

### Resource 1

Download URL: www.ncbi.nlm.nih.gov/sra/?term=PRJNA1415251

Resource identifier: PRJNA1415251

Data format : FASTQ

### Resource 2

Download URL: www.ebi.ac.uk/ena/browser/view/PRJEB85273

Resource identifier: PRJEB85273

Data format : FASTQ

## Usage Rights

Creative Commons Attribution 4.0 International (CC-BY-4.0)

Usage Rights

## Supplementary Material

6B97A495-8141-5667-B20A-89A803D517E5 10.3897/BDJ.14.e187301.suppl1 Supplementary material 1 1^st^-step PCR primers with diversity spacers used for the amplification of the 16S rRNA gene. Data type primer sequence table Brief description For each primer, the name, direction (Forward: F or Reverse: R) and sequence are provided. Bold letters denote the diversity spacers. File: oo_1281129.docx https://binary.pensoft.net/file/1281129 Jon Bent Kristofferssen

485669AC-A75A-5CBF-83FC-C427852B2F3F 10.3897/BDJ.14.e187301.suppl2 Supplementary material 2 Summary statistics for the 16S rRNA gene Illumina data. Data type sequencing statistics Brief description For each submitted and publicly available fastq file, the percentage (%) of reads containing primers and adapters at both ends is shown, along with the corresponding percentage following cleaning with the cutadapt software. File: oo_1601679.tsv https://binary.pensoft.net/file/1601679 Anastasia Gioti

101EAD28-A8F4-5C1A-A966-9CC7BCA26E5A 10.3897/BDJ.14.e187301.suppl3 Supplementary material 3 Quality of ONT data used for identification of fungal taxa. Data type quality scores, sequence lengths Brief description Average quality scores at different read lengths (central panel), density of read lengths (upper panel) and density of average quality scores (right panel) for the ONT data following processing with the proname_filter module. File: oo_1523337.png https://binary.pensoft.net/file/1523337 Bekir Kaşlı

D0FE09BE-1639-5282-BE47-F9643064B6F8 10.3897/BDJ.14.e187301.suppl4 Supplementary material 4 Summary statistics on the 8 custom-made training datasets tested with the RDP classifier. Data type sequence dataset statistics Brief description For each of the eight sequence datasets used for classifier training, the number of sequences, including unique sequences per taxon, along with the number of taxa at different taxonomic levels is shown, along with AUC, specificity and sensitivity at bootstrap 80. The above were used as criteria for selection of the optimal dataset for RDP training. File: oo_1565557.tsv https://binary.pensoft.net/file/1565557 Anastasia Gioti, Bekir Kaşlı

DE8791F6-28D5-5C6F-B213-DEB75998A225 10.3897/BDJ.14.e187301.suppl5 Supplementary material 5 RDP classifier training with different models for the identification of fungal taxa. Data type occurrences, model performance data Brief description A) Taxa count distribution at the genus-level (upper panel) and species-level (lower panel) for the custom datasets used in the RDP classifier training. B) Comparison of the four datasets in terms of Area Under the Curve values at different taxonomic ranks. File: oo_1523300.png https://binary.pensoft.net/file/1523300 Bekir Kaşlı - Anastasia Gioti

D4A44872-76D7-51CB-9442-6B1703470475 10.3897/BDJ.14.e187301.suppl6 Supplementary material 6 Metadata associated with publicly accessible data resources of the present study. Data type accession numbers, url, other metadata Brief description For each sample, correspondence to its associated Bioproject, Biosample, and run accession number with download links is presented, along with information on the most relevant experimental treatment conditions. File: oo_1601680.tsv https://binary.pensoft.net/file/1601680 Anastasia Gioti
