# Supplementary material for: Microbial communities associated with two populations of the sponge Chondrilla nucula under present and projected climate conditions in the Aegean Sea
Source: Biodivers Data J. 2026 Apr 29;14:e187301. doi: 10.3897/BDJ.14.e187301 (PMC13150538; doi:10.3897/BDJ.14.e187301)
Supplement: Supplementary material 1 — 1st-step PCR primers with diversity spacers used for the amplification of the 16S rRNA gene. [file bdj-14-e187301-s001.docx]

| Primer name | Direction | Sequence: Tail - spacer- specific primer |
| --- | --- | --- |
| 341F_00 | F | ACACTCTTTCCCTACACGACGCTCTTCCGATCT CCTACGGGNGGCWGCAG |
| 341F_01 | F | ACACTCTTTCCCTACACGACGCTCTTCCGATCT **A** CCTACGGGNGGCWGCAG |
| 341F_02 | F | ACACTCTTTCCCTACACGACGCTCTTCCGATCT **TG** CCTACGGGNGGCWGCAG |
| 341F_03 | F | ACACTCTTTCCCTACACGACGCTCTTCCGATCT **TAA** CCTACGGGNGGCWGCAG |
| 341F_04 | F | ACACTCTTTCCCTACACGACGCTCTTCCGATCT **CTGT** CCTACGGGNGGCWGCAG |
| 341F_05 | F | ACACTCTTTCCCTACACGACGCTCTTCCGATCT **GTTAT** CCTACGGGNGGCWGCAG |
| 341F_06 | F | ACACTCTTTCCCTACACGACGCTCTTCCGATCT **AGAGGT** CCTACGGGNGGCWGCAG |
| 805RB1_00 | R | GTGACTGGAGTTCAGACGTGTGCTCTTCCGATCT GACTACNVGGGTATCTAATCC |
| 805RB1_01 | R | GTGACTGGAGTTCAGACGTGTGCTCTTCCGATCT **G** GACTACNVGGGTATCTAATCC |
| 805RB1_02 | R | GTGACTGGAGTTCAGACGTGTGCTCTTCCGATCT **TG** GACTACNVGGGTATCTAATCC |
| 805RB1_03 | R | GTGACTGGAGTTCAGACGTGTGCTCTTCCGATCT **ATA** GACTACNVGGGTATCTAATCC |
| 805RB1_04 | R | GTGACTGGAGTTCAGACGTGTGCTCTTCCGATCT **CATA** GACTACNVGGGTATCTAATCC |
| 805RB1_05 | R | GTGACTGGAGTTCAGACGTGTGCTCTTCCGATCT **TCGTA** GACTACNVGGGTATCTAATCC |

Supplementary table 1. 1^st^-step PCR primers used in this study.
