# Supplementary figures and images for: Microbial communities associated with two populations of the sponge Chondrilla nucula under present and projected climate conditions in the Aegean Sea
Source: Biodivers Data J. 2026 Apr 29;14:e187301. doi: 10.3897/BDJ.14.e187301 (PMC13150538; doi:10.3897/BDJ.14.e187301)

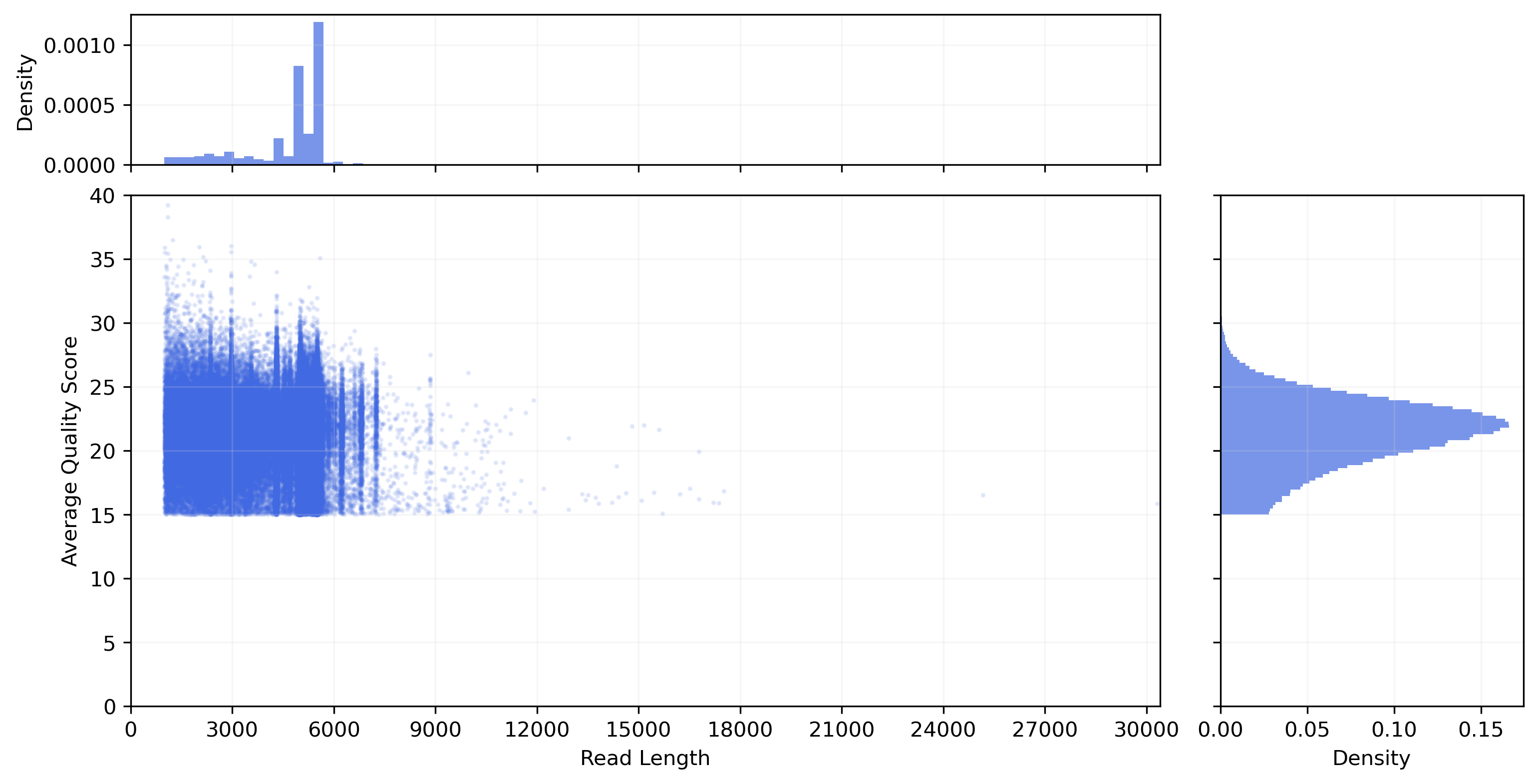

Supplement: Supplementary material 3 — Quality of ONT data used for identification of fungal taxa. [file bdj-14-e187301-s003.png]

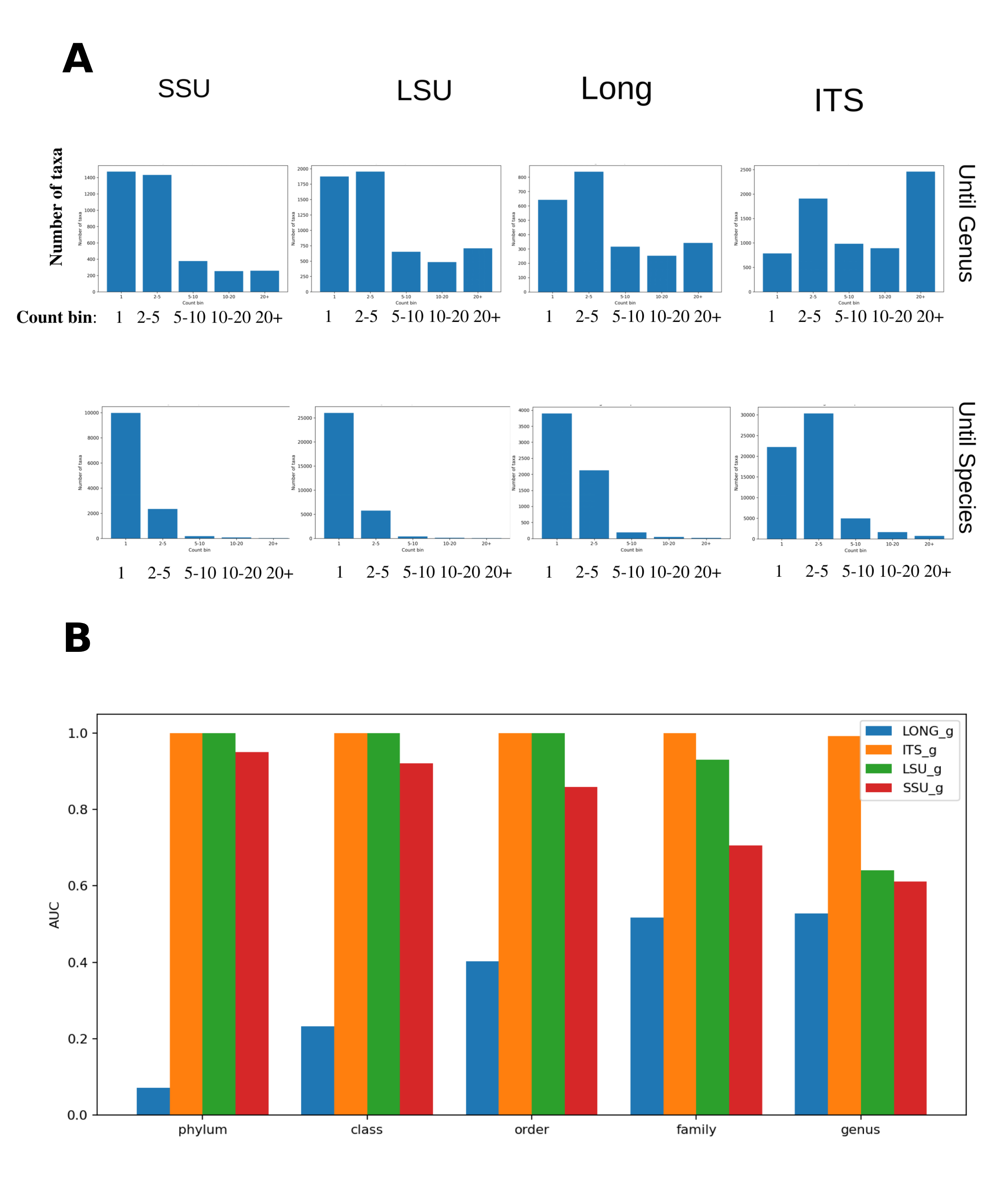

Supplement: Supplementary material 5 — RDP classifier training with different models for the identification of fungal taxa. [file bdj-14-e187301-s005.png]
